# From Perceived Vulnerability to Disease to Psychological Distress in times of COVID19 pandemic

**DOI:** 10.1192/j.eurpsy.2022.682

**Published:** 2022-09-01

**Authors:** A.T. Pereira, C. Cabacos, A. Araújo, A. Manão, A.P. Amaral, M.J. Soares, R. De Sousa, A. Macedo

**Affiliations:** 1 Faculty of Medicine of University of Coimbra, Institute Of Psychological Medicine, Coimbra, Portugal; 2 Centro Hospitalar e Universitário de Coimbra, Department Of Psychiatry, Coimbra, Portugal; 3 University Beira Interior, Faculty Of Health Sciences, Covilhã, Portugal; 4 Coimbra Institute for Biomedical Imaging and Translational Research, Icnas, Coimbra, Portugal

**Keywords:** disease, COVID19 pandemic, psychological distress, vulnerability

## Abstract

**Introduction:**

Perceived vulnerability to disease/PVD may influence psychological reactions to COVID-19 pandemic.

**Objectives:**

To analyse the role of PVD in psychological distress/PD during the COVID-19 pandemic, testing whether it is mediated by perceived risk of COVID-19, fear of COVID-19 and repetitive negative thinking/RNT.

**Methods:**

Participants (N=413 adults; 69.2% women) were recruited from September until December 2020, via social networks. They completed the following self-report validated questionnaires: Perceived Vulnerability to Disease Questionnaire/PVDQ; Perceived Risk of COVID-19 Scale, Fear of COVID-19 Scale; Perseverative Thinking Questionnaire and Depression Anxiety and Stress Scale. As women had significantly higher levels of PVD, COVID-19 perceived risk and fear, RNT, and psychological distress/PD, gender was controlled in mediation analysis (using PROCESS macro for SPSS; Hayes 2018).

**Results:**

All the variables significantly (p<.01), moderately (r>.20) and positively correlated. The serial mediation model 6 with the three sequential mediators resulted in significant total effect (c=.326, se=.0791, p<.001, CI:.1702-.4814), non-significant direct effect (c’=.111, se=.065, p=.087, CI:-.0162 to .2380), significant total indirect effect (.2149, se=.065, CI:.1079-.3278); most indirect effects were significant, including the indirect 7 (.0144, se=.0077, CI=.0017-.0320), that goes through all mediators (PVD->COVID19 perceived risk->COVID19 fear->RNT->PD), meaning full mediation.

**Conclusions:**

The effect of PVD on psychological distress operates by increasing the perception of risk and the fear of COVID-19, which intensify related worries and ruminations in times of pandemic. People with high perceived threat, aversion and discomfort in situations associated with increased risk of infection should be helped to decrease dysfunctional cognitive contents and processes in times of pandemic.

**Disclosure:**

No significant relationships.

